# Food-derived molecules as regulators of intestinal tight junctions and barrier function: mechanisms and implications

**DOI:** 10.3389/fddev.2026.1692219

**Published:** 2026-03-20

**Authors:** Sinéad M. Ryan, David J. Brayden

**Affiliations:** School of Veterinary Medicine and Conway Institute, University College Dublin, Belfield, Dublin, Ireland

**Keywords:** butyrate, fermented foods, food-derived bioactive molecules, GI tract, gut microbiome, intestinal epithelial permeability, intestinal tight junction regulation, short chain fatty acid

## Abstract

Controlling TJ permeability in the small intestine facilitates nutrient absorption, maintains luminal osmotic balance, and prevents the paracellular entry of pathogens. The pharmaceutical industry has leveraged the capacity of medium-chain fatty acids and their derivatives to transiently and reversibly open epithelial TJs in formulations to enable oral administration of therapeutic peptides, some of which have received regulatory approval or are progressing in advanced clinical trials. Other food-derived agent including chitosan and its analogues enhance mucoadhesion and also modulate TJ permeability in the intestine. Recently, pelargonidin, a polyphenolic pigment isolated from strawberries, has emerged as a promising food-derived TJ opener, facilitating oral insulin delivery in rat models. Conversely, other food or food-derived molecules reinforce TJ integrity while exerting antioxidant effects, thereby offering potential therapeutic benefits for conditions characterized by increased intestinal permeability including inflammatory bowel disease, sepsis, and coeliac disease. Examples of such agents include the short-chain fatty acid (SCFA), sodium butyrate, various essential and non-essential amino acids, fermented food, the trace element, zinc, and anthocyanins. The exploration of food-derived substances as modulators of intestinal epithelial TJ dynamics is still in its early stages but holds significant promise for future health applications.

## 1 Introduction

The intestinal barrier is essential for maintaining equilibrium between the gut lumen and the internal milieu of the body (Vancamelbeke and Vermeire, 2017). The intestinal epithelium functions as a selectively permeable interface, enabling the transcellular and paracellular transport of key nutrients, electrolytes, and water, while simultaneously restricting the entry of potentially harmful substances such as food antigens, digestive enzymes, endotoxins, pathogenic microorganisms, and their associated toxins. This selective permeability is vital for maintaining intracellular and systemic homeostasis. The Hippocratic quote, “All disease begins in the gut,” while perhaps overstated, reflects a growing appreciation for the gut’s central role in health and disease. The interplay between the intestinal barrier and the gut microbiota has become a major focus of research, not only in understanding disease mechanisms but also in the development of novel strategies for oral therapeutic delivery (Inczefi et al., 2022). Compromised TJ integrity is increasingly recognized as a hallmark of pathological conditions including gastrointestinal infections, inflammatory bowel disease (IBD), food allergies, autoimmune disorders, certain gastrointestinal cancers, and metabolic syndromes (Ciorba et al., 2024; Longo et al., 2024). Despite this, temporarily and reversibly increasing intestinal permeability with permeation enhancers (PE) has proved a beneficial strategy in creating oral formulations of several therapeutic peptides that have been approved by the regulatory authorities for systemic delivery (Brayden and Maher, 2021; Lewis et al., 2022). Thus, maintaining a functional intestinal barrier is fundamental to both gastrointestinal and systemic health.

## 2 The intestinal barrier

### 2.1 Structure of intestinal barrier

**FIGURE 1:**
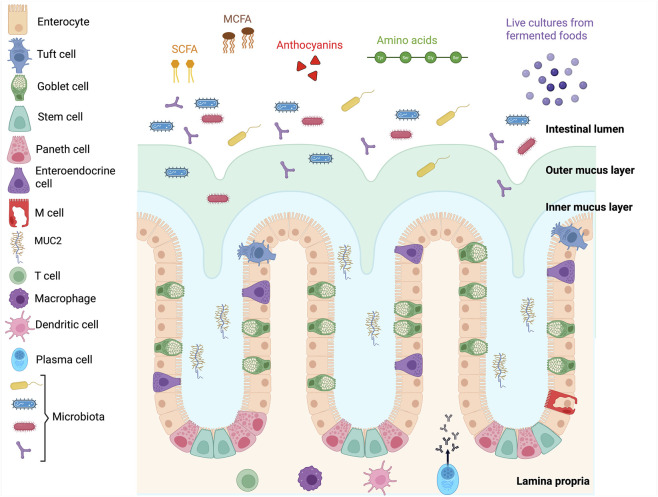
Schematic diagram of the different layers and components of a healthy intestinal barrier. Created in BioRender (https://BioRender.com/6mil1kz).

The intestinal barrier is a highly specialized and dynamic structure that regulates the selective absorption of essential nutrients, electrolytes, and water, while simultaneously protecting the host from luminal pathogens, toxins, and antigens (Zhang et al., 2023). It comprises several interdependent components that work in concert to maintain GI homeostasis and immune surveillance (Figure 1). A key non-host element of this barrier is the gut microbiome, a complex and diverse community of bacteria, fungi, viruses, and archaea that resides within the intestinal lumen. These microorganisms contribute to digestion, produce essential metabolites such as short-chain fatty acids like butyrate, acetate, and propionate which are key energy sources for gut cells. It also modulates immune responses, and competitively inhibit the colonization of pathogenic species (Ford, 2025). Together, the gut microbiota and epithelial tight junctions form the intestinal barrier which is a critical defence system that maintains selective permeability. Within this system, microbial signalling to epithelial cells modulates tight junction proteins, thereby regulating gut permeability to permit nutrient absorption while preventing pathogen translocation. This balanced ecosystem resists colonization by pathogens and helps maintain metabolic and inflammatory homeostasis, contributing to overall gastrointestinal and systemic health.

The next structural layer is the mucus barrier, composed of a hydrated gel rich in highly glycosylated mucins secreted by goblet cells (Pelaseyed et al., 2014). This layer physically separates luminal contents from the epithelium and serves as a diffusion barrier for macromolecules. Mucins are broadly categorized as secreted gel-forming mucins, such as MUC2, which constitute the main framework of the mucus layer, and transmembrane mucins, including MUC1, which form part of the glycocalyx covering epithelial cells (Paone and Cani, 2020). Beneath the mucus lies a monolayer of polarized epithelial cells, organized into crypt-villus units that line the luminal surface (Barrett, 2020). This epithelium functions as a selective physical barrier, tightly regulated by TJ complexes that connect adjacent cells and restrict paracellular flux. The intestinal epithelium comprises several specialized cell types, each contributing uniquely to gut homeostasis and defence. Enterocytes are the primary absorptive intestinal epithelial cells, responsible for the uptake of nutrients and electrolytes, as well as the secretion of antimicrobial peptides. Goblet cells produce mucins that sustain the protective mucus layer covering the epithelium. Paneth cells, located at the base of the crypts, secrete antimicrobial proteins such as lysozyme and defensins, which help regulate the microbial environment. Enteroendocrine cells release hormones involved in nutrient sensing and gastrointestinal motility, notably glucagon-like peptides GLP-1 and GLP-2 (Brubaker, 2022). Microfold (M) cells, situated in Peyer’s patches, facilitate the uptake of luminal antigens and play a critical role in initiating mucosal immune responses (Park et al., 2023). Finally, tuft cells are rare chemosensory epithelial cells that contribute to Type 2 immune responses, particularly during parasitic infections (Pelaseyed et al., 2014). Beneath the epithelial monolayer lies the basement membrane (*lamina basalis*), a thin sheet of extracellular matrix composed of collagen, laminins, and proteoglycans. This layer provides structural support and facilitates epithelial–mesenchymal interactions. The underlying lamina propria contains blood and lymphatic vessels, as well as a rich population of immune cells, including macrophages, dendritic cells, T cells, and IgA-secreting plasma cells that together support mucosal immunity.

### 2.2 Challenges in oral delivery of macromolecules

Despite its protective role, the intestinal epithelial barrier presents a major obstacle to oral delivery of macromolecular therapeutics, particularly proteins and peptides. The hydrophilic nature and large molecular weight of these molecules prevent passive diffusion across enterocytes, and paracellular transport is restricted by TJ complexes, which typically exclude molecules larger than ∼1 nm or 600 Da (Shen et al., 2011; Navia and Chaturvedi, 1996). To address this, intestinal PEs based primarily on medium chain fatty acid (MCFA) structures have been investigated to transiently and reversibly increase paracellular permeability (Brayden et al., 2020). These compounds facilitate the absorption of poorly permeable drugs, including peptide therapeutics, by modulating TJ integrity and/or fluidizing the plasma membrane. Sodium caprate (C_10_), for example, acts via multimodal mechanisms to enhance TJ permeability and increase mucosal uptake of peptides (Maher et al., 2023). Other PEs including Sodium N-(8-[2-hydroxybenzoyl] amino) caprylate, also known as Salcaprozate Sodium (SNAC), Ethylenediaminetetraacetic acid (EDTA), bile salts, and other medium-chain fatty acids, have been incorporated into oral peptide formulations, with SNAC enabling the successful Food and Drug Administration (FDA) approval of oral semaglutide tablets (RYBELSUS®). Nevertheless, the clinical translation of many PEs remains limited. Some PEs have been discontinued due to cytotoxicity or structural damage to the intestinal mucosa (McCartney et al., 2016; Maher et al., 2009a). The low oral bioavailability typically achieved (∼1%), a value that restricts the feasibility of oral delivery to only a few highly potent peptides (Brown et al., 2020). Despite these challenges, continued research in this area holds promise for expanding the oral delivery landscape of biologics and improving patient compliance (Bohley and Leroux, 2024). Likewise, there are similar and additional limitations in translating food-derived macromolecules into clinical therapies administered by the oral route. Many are also peptides which have the same stability and intestinal permeability problems as the therapeutic ones emerging from Discovery programmes in Pharma companies. Moreover, food-derived peptides offering potential health benefits ultimately need to be produced in pure form at GMP grade and formulated with excipients in conventional solid dosage forms made at scale to provide precise doses for patients to produce the required pharmacodynamic outputs. It is unlikely that positive outcomes will be achieved or proven by re-packaging such macromolecules in adapted foods or drinks as the required dose levels, stability, and presentation at the gut wall have not been optimised.

## 3 Tight junction structure and function

**FIGURE 2:**
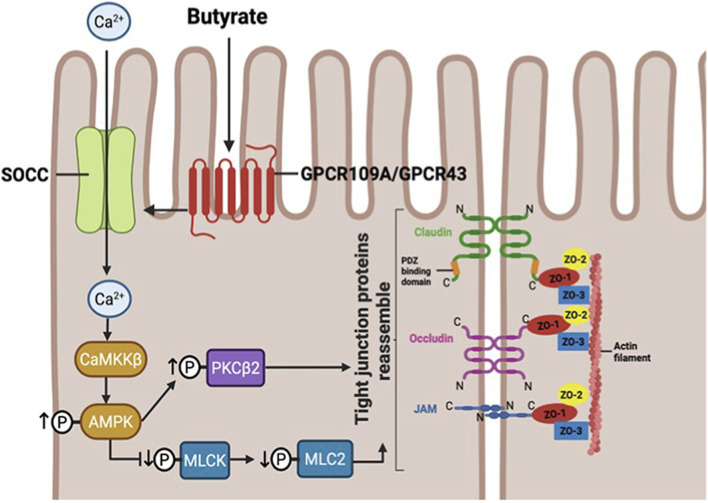
Schematic representation of the tight junction (TJ) complex in the intestinal epithelium and the proposed mechanism of butyrate-mediated TJ reassembly. Tight junctions are multi-protein complexes located at the apical region of the lateral membrane of intestinal epithelial cells. The TJ complex consists of both transmembrane and intracellular scaffold proteins. Transmembrane components include claudins, occludin, and junctional adhesion molecules (JAMs) which form a permselective barrier that regulates paracellular permeability. Their intracellular domains interact with scaffold proteins of the zonula occludens (ZO) family (ZO-1, ZO-2, and ZO-3), which anchor the transmembrane proteins to the actin cytoskeleton, providing structural stability. Butyrate binds to G-protein-coupled receptors (GPCRs), leading to activation of store-operated calcium channels (SOCCs) and subsequent Ca^2+^ influx. This rise in intracellular calcium activates CaMKKβ, which in turn activates AMP-activated protein kinase (AMPK). AMPK activation promotes downstream signalling through protein kinase C β2 (PKCβ2) and inhibition of the myosin light chain kinase (MLCK)/myosin light chain 2 (MLC2) pathway, ultimately facilitating tight junction reassembly and barrier restoration. Created in BioRender (https://BioRender.com/ay25y69).

TJs are intracellular junctions in epithelial cells that regulate the integrity of the epithelial barrier and contribute to cellular polarity. They are a multifunctional complex that forms a seal between adjacent epithelial cells near the apical surface (Citi et al., 2024). They seal the paracellular space between epithelial cells, thus preventing paracellular diffusion of microorganisms and other antigens across the epithelium. TJs are highly dynamic structures that are constantly being remodelled due to interactions with external stimuli, such as food residues and pathogenic and commensal bacteria. They can regulate the entry of nutrients, ions, and water while restricting pathogen entry and thus control the barrier function of the epithelium. TJs are formed by the interaction of transmembrane proteins including claudins, occludins, and junctional adhesion molecules (JAMs), along with the cytoplasmic scaffolding proteins, zonula occluden-1 (ZO-1), ZO-2, and ZO-3 (Figure 2). The following is a current description of how these transmembrane proteins regulate epithelial barrier function and polarity.

### 3.1 Claudins

Claudins are integral membrane proteins that play a pivotal role in maintaining the integrity and selective permeability of TJs within the intestinal epithelium. These proteins are essential for regulating the paracellular transport thereby controlling the selective passage of ions, solutes, and water between intestinal epithelial cells (Van Itallie and Anderson, 2014), maintaining cell polarity and facilitating cell differentiation and proliferation (Farkas et al., 2012). Claudins are a family of tetraspan proteins, each characterized by four transmembrane domains, two extracellular loops, and cytoplasmic N-terminal and C-terminal ends. The first extracellular loop is notably larger than the second and contains charged amino acids that determine the protein’s charge selectivity and barrier properties. These extracellular adhesion points are the most targetable TJ component for modulation of the paracellular barrier (AlMarzooqi et al., 2024). The C-terminal domain includes a PDZ-binding motif, facilitating interactions with scaffold proteins such as ZO proteins, which anchor claudins to the actin cytoskeleton.

Within the intestinal tract, various claudin isoforms are expressed in a segment-specific manner, contributing to the unique barrier properties of selected regions. In the context of the intestinal epithelium, 24 claudin members have been identified in mammals, but claudin 13 is missing in humans. Some claudins act in part by controlling barrier function and paracellular permeability (e.g., claudin-2, -7 and -12), while others control barrier properties such as electrical tightness of the junctions (claudin-1, -3, -4, -5 and -8) (Barmeyer et al., 2015). These claudins can also be categorised based on selectivity: anion selective (claudin-17), cation selective (claudin-15), water selective (claudin-2), while others exhibit barrier properties regardless of charge (claudin-1, -3 and -5). Several claudin knockout mouse models have been generated, and their diverse phenotypes clearly demonstrate the importance of claudin proteins in maintaining tissue integrity and homeostasis in various organs (Ding et al., 2012; Seker et al., 2019; Xu et al., 2019; Ding et al., 2022). Claudin-7 is expressed in epithelia of both the small and large intestine. Using Claudin-7–deficient mouse models, Xu et al. demonstrated a critical role for Claudin-7 in maintaining intestinal epithelial homeostasis. While inducible conditional Claudin-7 knockout mice exhibited normal development at birth, acute deletion of Claudin-7 in adulthood led to rapid deterioration and a moribund phenotype. Histopathological analysis revealed severe intestinal inflammation accompanied by atypical epithelial hyperplasia and adenoma formation. Consistent with these findings, aberrant Ki67 expression and distribution indicated a profound disruption of normal epithelial proliferative control. Ki67 is a protein that is found only in cells that are dividing. A high Ki-67 proliferation index means many cells are dividing quickly and that the cancer is likely to grow and spread (Liu et al., 2024; Krug et al., 2014). Collectively, these results highlight Claudin-7 as an essential regulator of intestinal barrier integrity and epithelial proliferation, and suggest that its loss predisposes the intestine to inflammation-driven dysplasia and tumorigenesis (Xu et al., 2019).

Claudin-4 is a barrier-forming tight junction protein in the intestinal epithelium that strengthens transepithelial resistance by limiting paracellular ion flux and inhibiting the channel activity of pore-forming claudins to fine-tune barrier function. Targeting claudin-4 has emerged as a strategy for modulating epithelial permeability. For example, the peptide PN159 (KLAL/MAP) is reported to bind claudin-4, destabilizing tight junctions and thereby increasing paracellular permeability (Bocsik et al., 2019). Interestingly, Shashikanth et al. identified ‘interclaudin interference’, a post-translational mechanism whereby one claudin disrupts the structure and ion channel activity of another. They used claudin-4 as a model barrier-forming claudin. In MDCK I monolayers, claudin-4 alone neither formed nor markedly strengthened the paracellular barrier, but when co-expressed with pore-forming claudins (2, 7, 15, 19), it collapsed claudin meshworks, depolymerized strands, and inhibited cation channel function, thereby enhancing barrier integrity. This mechanism enables rapid modulation and fine-tuning of paracellular permeability (Shashikanth et al., 2022). Although the underlying mechanisms of claudin regulation in various tissues and their exact roles in normal physiology as well as in disease states are being elucidated, more research work remains to be done (Wang et al., 2024; Jiang et al., 2019; Brunner et al., 2021).

### 3.2 Occludin

Occludin was the first TJ protein identified (Farquhar and Palade, 1963). They are tetraspanin integral membrane proteins with four transmembrane domains and cytoplasmic N- and C-termini. Two extracellular loops of occludin, rich in glycine and tyrosine residues, interact with neighbouring occludins on adjacent cells, contributing to the paracellular barrier by restricting macromolecule flux while allowing small ion passage (Al-Sadi et al., 2011). The C-terminal domain interacts with the scaffold proteins, ZO-1, ZO-2, and ZO-3, linking occludin to the actin cytoskeleton and anchoring it within the TJ complex (Furuse et al., 1994). While claudins form the ion-selective pores of TJs, occludin is more closely associated with sealing and regulatory functions, including control of the “leak pathway” for larger molecules (Al-Sadi et al., 2011; Balda et al., 2000; Feldman et al., 2005; Buschmann et al., 2013). Occludin expression levels correlate with epithelial barrier integrity both *in vitro* and *in vivo* (Balda et al., 2000; Singh et al., 2020; Monaco et al., 2021), and its loss is linked to increased permeability under inflammatory conditions.

Inflammatory cytokines such as IL-1β downregulate occludin expression selectively *in vitro* and *in vivo*. This occurs via miRNA-mediated post-transcriptional regulation; IL-1β induces miR-200c-3p, which binds to the 3′-UTR of occludin mRNA, reducing its expression and increasing TJ permeability (Rawat et al., 2020). Inhibition of miR-200c-3p restores occludin levels and barrier function. Functional studies further implicate occludin in maintaining epithelial architecture. Transfection of the oncogene c-RAF in rat parotid epithelial cells results in loss of TJ integrity, downregulation of occludin and claudin-1, and altered ZO-1 and E-cadherin localization. Reintroduction of human occludin reverses many of these changes, including restoration of monolayer morphology, claudin-1 expression, and partial recovery of transepithelial electric resistance (TEER) (Li and Mrsny, 2000).

Although occludin knockout mice initially appeared to retain normal intestinal TJ function (Saitou et al., 2000; Schulzke et al., 2005), subsequent studies have identified physiological defects including chronic gastritis, deafness, male infertility, and neuroinflammation, which supports a broader role for occludin in epithelial and endothelial barrier maintenance (Kitajiri et al., 2014; Liu et al., 2022). In intestinal inflammation, reduced occludin expression is frequently observed in patient biopsies from IBD. However, its loss may also serve as an adaptive rather than causal mechanism. In murine colitis models, epithelial-specific occludin knockout attenuated disease severity by conferring resistance to apoptosis via suppression of caspase-3 expression. Promoter analysis revealed occludin enhances caspase-3 transcription, and its downregulation promotes epithelial survival under inflammatory stress (Kuo et al., 2019). Thus, occludin may play a dual role: supporting barrier integrity under homeostatic conditions but allowing protective adaptation in inflamed tissue.

### 3.3 Zonula occludens

ZO proteins are essential scaffolding proteins in the intestinal barrier. They are part of the membrane-associated guanylate kinase (MAGUK) family and are essential for organizing and stabilizing TJ complex. ZO proteins are multidomain proteins that bridge the integral membrane proteins of TJs (such as claudins, occludins, and JAMs) and the actin cytoskeleton. Three ZO proteins, ZO-1 (∼220 kDa) (Stevenson et al., 1986), ZO-2 (∼160 kDa) (Gumbiner et al., 1991), and ZO-3 (∼130 kDa) (Haskins et al., 1998), have been identified to date. They are multidomain proteins with three PSD95-DIgA-ZO-1 (PDZ) domains, a central Src homology 3 (SH3) domain, and a region with homology to guanylate kinase (GUK) (Van Itallie and Anderson, 2014) and actin-binding regions, which enable them to organize TJ components and link them to the cytoskeleton. The PDZ domains bind to the C-terminal tails of claudins, occludins, and JAMs, anchoring them to the TJ complex (Ebnet et al., 2000). The SH3 domain mediates interactions with signalling proteins and other cytoskeletal components. The GUK domain, although enzymatically inactive in ZO proteins, serves as a binding site for other proteins, including transcription factors and signalling molecules. ZO proteins also have a region that binds directly to actin filaments, linking the TJ complex to the cytoskeleton. The indirect interaction of ZO proteins with the cytoskeleton involves several actin-binding proteins including cortactin (Katsube et al., 1998), alpha-catenin (Muller et al., 2005), protein 4.1R (Mattagajasingh et al., 2000), the Ras target, AF6/afadin, as well as the actin- and myosin-binding proteins, cingulin (D'Atri et al., 2002) and Shroom (Etournay et al., 2007). This interaction is crucial for maintaining the structural integrity and dynamic regulation of TJs.

In order to gain more insight into the functional significance of ZO proteins, ZO deficient/knockout cell and mouse models have been used. ZO-1 and ZO-2 were shown to be essential in TJ assembly, because ZO-1 or ZO-2-deficient Eph4 cells (spontaneously immortalized mouse mammary gland epithelial cells) failed to form TJs (Umeda et al., 2006; Phua et al., 2014). Umeda et al., have shown that ZO-1-deficient cells are still able to form normal TJ structures and show normal permeability; however, a delay in barrier establishment of other TJ proteins including occludin and claudins in the TJ was observed, indicating that ZO proteins have a role to play in the regulation of TJ assembly (Umeda et al., 2004). Similar results were obtained by McNeil et al. showing that knockdown of ZO-1 in MDCK cells retarded TJ formation (McNeil et al., 2006). It also appears that ZO-1 mediates early assembly of TJ proteins in bilateral cells (Tornavaca et al., 2015). The first PDZ domain of ZO-1 interacts with claudin proteins, the second domain interacts with other ZO proteins (ZO-1, -2 and -3), and the third domain interacts with JAM-A (Ulluwishewa et al., 2011). Due to this complex interaction ZO-1 plays a central role in TJ regulation and binds directly to F-actin (a cytoskeleton protein).

Kuo et al. assessed the contributions of ZO-1 to epithelial barrier function and mucosal homeostasis (Kuo et al., 2021). They found the ZO-1 transcript and protein expression were reduced in IBD patient biopsies. *In vivo* studies showed that despite slightly increased intestinal permeability, intestinal epithelial-specific ZO-1 knockout mice were healthy and did not develop spontaneous disease. However these ZO-1 knockout mice were hypersensitive to mucosal challenges and displayed defective repair. Furthermore, ZO-1-deficient colonic epithelia failed to upregulate proliferation in response to damage *in vivo* or Wingless-related integration site (Wnt) signalling *in vitro*. ZO-1 associated with centrioles in interphase cells and mitotic spindle poles during division. In the absence of ZO-1, mitotic spindles failed to correctly orient, resulting in the incompletion of mitosis. ZO-1 is therefore critical for upregulation of epithelial proliferation and mucosal repair (Kuo et al., 2021).

### 3.4 Junctional adhesion molecules

JAMs are a family of proteins that belong to the immunoglobulin (Ig) superfamily. They are primarily located at cell-cell junctions, including TJs, which are critical for maintaining the integrity of epithelial and endothelial barriers. The JAM family includes JAM-A, JAM-B, JAM-C and JAM-4. The JAM family are comprised of extracellular Ig domains, a transmembrane domain, and an intracellular C-terminal domain (Otani and Furuse, 2020). JAMs have two extracellular immunoglobulin-like domains, a V-type (variable-like) domain followed by a C2-type (constant-like) domain. These domains are involved in homophilic (JAM-JAM) and heterophilic (JAM-other proteins) interactions. A single-pass transmembrane domain anchors the protein in the cell membrane (Steinbacher et al., 2018). The intracellular C-terminal domain of JAMs is relatively short and contains binding sites for various intracellular proteins including those involved in signalling and cytoskeletal organization. For example, JAM-A has a PDZ-binding motif at its C-terminus, which allows it to interact with PDZ domain-containing proteins like ZO-1. JAM members are expressed in various cell types including epithelial, endothelial, and immune cells. In intestinal epithelial cells, JAM-A, JAM-4, and coxsackievirus and adenovirus receptors are expressed and involved in TJ regulation. However the role of JAM-4 in TJs is still unclear (Hartmann et al., 2020; Hirabayashi et al., 2003). The first indications for a role of JAM-A in epithelial barrier formation *in vivo* came from observations in patients suffering from intestinal inflammatory disorders such as Crohn’s disease (CD) or ulcerative colitis (UC), which represent the two major forms of IBD (Vetrano et al., 2008). JAM-A are concentrated at epithelial and endothelial TJs, and the extracellular N-terminal domains bind to ligands through hemophilic (formation of TJs and bicellular border) and heterophilic interactions (Fan et al., 2019). JAM-A plays a significant role in intestinal homeostasis and epithelial permeability. In several CD and UC patients the levels of JAM-A in the colon were reduced, correlating reduced JAM-A expression with impaired mucosal barrier function (Vetrano et al., 2008). Also, when colitis is induced experimentally in mice using dextran sodium sulphate (DSS), JAM-A expression levels are strongly reduced when compared to controls (Vetrano et al., 2008). Other studies have also shown that JAM-A knockout mice exhibit higher permeability to dextran and myeloperoxidase activity in the colon compared to wild type mice. In addition, the colonic injury and inflammation induced by DSS are more severe in JAM-A knockout mice than in wild type mice (Laukoetter et al., 2007). JAM-A orchestrates the spatiotemporal association of scaffold molecules such as ZO-2 and Afadin that associate with Rap activators such as PDZ-GEFs to modulate activity of small GTPases in the Rap and Rho family, in order to regulate barrier function and integrin-dependent cell migration both *in vitro* and *in vivo* (Monteiro et al., 2013). Fan et al., showed that tyrosine phosphorylation of JAM-A Y280 destabilized TJ-associated signalling elements, which resulted in decreased Rap2 GTPase activity and compromised epithelial barrier. They observed increased phosphorylation of Y280 in colonic epithelium of individuals with UC and in mice with experimentally induced colitis (Fan et al., 2019). These findings have provided important insights into how epithelial-expressed JAM-A regulates intestinal permeability. JAM-A also prevents the development of metabolic dysfunction-associated steatotic liver disease (MASLD). There is evidence that a loss of the intestinal barrier function contributes to MASLD (Anstee et al., 2019). JAM-A-deficient mice fed with a diet high in fat, fructose and cholesterol develop all symptoms typical for MASLD including steatohepatitis, inflammation and fibrosis concomitant with increased intestinal epithelial permeability (Rahman et al., 2016). MASLD patients also have reduced levels of JAM-A in the intestinal mucosa (Rahman et al., 2016). Findings in JAM-A-deficient mice further establish an important role for JAM-A for the intestinal barrier.

Prolonged exposure of Caco-2 and SK-CO15 human intestinal epithelial monolayers to ethanol reduced JAM-A protein expression by 70%. Furthermore, acute exposure of Caco-2 cells to alcohol was associated with reduced Ras-related protein 2 (Rap2) activity, and enhanced myosin light chain kinase (MLCK) activity, changes consistent with impaired JAM-A signalling. Acute exposure of Caco-2 cells to alcohol was associated with perturbations of Rap2 and MLCK activity, signalling molecules regulated downstream of JAM-A, demonstrating the critical role of JAM-A in the regulation of paracellular permeability (Chopyk et al., 2017). JAM-A also promotes intestinal mucosal wound healing *in vivo* through regulation of a promigratory protein signalling complex between Rap1A, Talin, and β1 integrin (Fan et al., 2022).

### 3.5 Myosin light chain kinase and cytoskeletal regulation

Myosin light chain kinase (MLCK)–mediated regulation of epithelial barrier function is primarily controlled by coordinated Ca^2+^/calmodulin and RhoA/Rho-associated kinase (ROCK) signalling pathways that converge on phosphorylation of the regulatory myosin light chain (MLC-2), a central determinant of actomyosin contractility and TJ permeability (Cunningham and Turner, 2012). Increases in intracellular Ca^2+^, resulting from receptor-mediated influx or release from intracellular stores, promote Ca^2+^ binding to calmodulin, which induces a conformational change that relieves MLCK autoinhibition and activates its kinase function (Takashima, 2009; Garcia et al., 1995). Activated MLCK directly phosphorylates MLC-2 at Ser19 and/or Thr18, enhancing myosin ATPase activity and promoting actin–myosin cross-bridge cycling within the perijunctional actomyosin ring. In parallel, activation of the small GTPase RhoA stimulates ROCK, which further increases MLC-2 phosphorylation both directly and indirectly by inhibiting myosin light chain phosphatase (MLCP) through phosphorylation of its regulatory subunit MYPT1 and phosphatase inhibitors such as CPI-17 (Jin and Blikslager, 2020; Rezaei et al., 2024; Aguilar et al., 2012). The ROCK-dependent suppression of MLCP results in Ca^2+^ sensitization of the contractile apparatus, sustaining elevated MLC-2 phosphorylation even without persistent Ca^2+^ elevation. The phosphorylation state of MLC-2 therefore reflects a dynamic balance between MLCK-mediated phosphorylation and MLCP-mediated dephosphorylation, with increased phosphorylation generating ATP-dependent actomyosin contraction and elevated cytoskeletal tension at the apical junctional complex (Jin and Blikslager, 2020). In intestinal epithelial cells, this enhanced contractile tension induces centripetal contraction of the perijunctional actomyosin ring, leading to internalization and reorganization of junctional proteins, including claudins, occludin, ZO-1, and adherens junction components, resulting in reversible TJ opening and increased paracellular permeability. Non-muscle MLCK isoforms, particularly MLCK1, are highly enriched at the perijunctional actomyosin ring in differentiated villous epithelium and play a dominant role in regulating physiological and pathological barrier dynamics, as selective MLCK1 inhibition or knockdown reduces junctional permeability (Jin and Blikslager, 2020). While tightly regulated MLCK and ROCK signalling supports normal epithelial transport and barrier modulation, excessive or sustained activation of these pathways contributes to pathological intestinal barrier dysfunction observed in inflammatory and infectious conditions (Bruewer et al., 2006; Chen et al., 2014; Xiong et al., 2026).

### 3.6 Leaky gut

Disruption of intestinal barrier integrity, commonly referred to as “leaky gut” has emerged as a key pathogenic factor in a wide range of chronic inflammatory and autoimmune diseases (Hollander and Kaunitz, 2020). Compromised TJ function leads to increased intestinal permeability, permitting abnormal translocation of luminal macromolecules, endotoxins, viruses, and lipopolysaccharides into the systemic circulation. This breach can trigger both local mucosal inflammation and systemic immune activation. Notably, elevated intestinal permeability has been implicated in IBD (Yu et al., 2022), celiac disease (Gundry, 2020) and Type 1 diabetes (Miele et al., 2020). Moreover, barrier dysfunction has been linked to diseases originating outside the GI tract, including IgA nephropathy (Tang et al., 2022b), MASLD (Tilg et al., 2022), and multiple sclerosis (Pellegrini et al., 2023). In severe cases, systemic entry of microbial products may lead to systemic inflammatory response syndrome with the potential for multi-organ failure (Ge et al., 2020). Importantly, intestinal barrier impairment can also occur in individuals without diagnosed GI disease. Experimental evidence indicates that epithelial permeability increases with age (Mullin et al., 2002). Other contributing factors include psychological stress and gut microbiota imbalances (Wang et al., 2022b), along with high alcohol intake (Turner et al., 2024; Swanson et al., 2024) and the use of nonsteroidal anti-inflammatory drugs (NSAIDs) (Bjarnason et al., 2018), both of which have been shown to transiently increase permeability.

While research has focused on pharmacological approaches to modulate the intestinal barrier, there is growing interest in the potential of food-derived molecules to influence TJ integrity and support gut health. Nutritional modulation of the intestinal barrier presents a promising strategy for disease prevention, management, and therapeutic delivery. The following sections focus on recent advances in the understanding of how food-derived components regulate intestinal TJ function, highlighting key categories of bioactive food compounds and their relevance to intestinal barrier integrity and health. A summary of food-derived components which have an effect on TJ permeability are listed in Table 1.

**TABLE 1:** Effect of food-derived compounds on intestinal permeability.

Food-derived compounds	Effect on permeability	Mechanism of TJ modulation	Model used	References
Short chain fatty acids				
Butyrate	Decreased	Enhances expression of Claudin 1,3 and 4	Rat model	Yan and Ajuwon (2017)
		Increases intracellular Ca^2+^ which activates AMPK	Caco-2 cells	Miao et al. (2016)
		Activation of PKCβ2	Caco-2 cells	Wang et al. (2023b)
		Stabilization of HIF through inhibition of iron-dependent prolyl hydroxylase enzymes	Mouse model	Ornelas et al. (2023)
Medium chain fatty acids				
Sodium caprylate (C_8_) (Mycapssa®)	Increased	Redistribution of ZO-1 and claudins	Phase III clinical trials	Melmed et al. (2015), Melmed et al. (2020)
Sodium caprate (C_10_)	Increased	Membrane fluidisation and intracellular calcium signalling cascades involving phospholipase C, IP_3_, and MLCK, which trigger cytoskeletal contraction and TJ opening	Caco-2 cells Phase IIb clinical trials	Tomita et al. (1996), Shimazaki et al. (1998), Ballantyne et al. (2023)
	Increased	Reversibly displaced tricellulin and claudin 5	Caco-2 cells	Krug et al. (2013)
Polysaccharides				
Chitosan	Increased under acidic conditions	Redistribution of ZO-1 and F-actin distribution interaction with apical integrins, activating downstream focal adhesion kinase and steroid receptor coactivators	Caco-2 cells	Hsu et al. (2012), Yeh et al. (2011)
N-Trimethyl chitosan	Increased	Activation of C-Jun NH_2_-terminal kinase-dependent pathway	Caco-2 cells	Huang et al. (2013)
MNA-TG-chitosan	Increased	Protein tyrosine phosphatase inhibition, reducing occludin dephosphorylation	TR-146 cells	Pratap-Singh et al. (2023)
Amino acids				
Glutamine	Decreased	Upregulation of claudin-1, occludin, and ZO-1 via ERK and NF-κB signalling pathways	Caco-2 cells	DeMarco et al. (2003), Li et al. (2004), Beutheu et al. (2013)
L-Tryptophan	Decreased	Upregulation of occludin, claudin-4, ZO-1 and 2	Porcine intestinal epithelial cells	Wang et al. (2015)
Tryptophan	Decreased	CaSR)/Ras-related C3 botulinum toxin substrate 1 (Rac1/phospholipase C gamma-1 (PLC-γ1) signalling pathway	Porcine intestinal epithelial cells	Liu et al. (2021a)
Aspartate, glutamate and glutamine	Decreased	Increase expression of occludin, claudin-1 and 3	Weaning piglets	Deng et al. (2023)
Minerals				
Zinc depletion	Increased	Downregulation of claudin-3 and occludin	Caco-2 cells	Camilleri (2021), Zhong et al. (2010)
Flavonoids				
Pelargonidin	Increased	Modulation of actin cytoskeletal dynamics	Mouse model	Lamson et al. (2022)
Anthocyanin	Decreased	Upregulation of occludin, ZO-1 and claudin-1	Mouse model	Cremonini et al. (2019)
Fermented foods				
Kefir	Potential decrease	Increase butyrate and propionate in the colon	Mouse model	Albuquerque Pereira et al. (2023), Choi et al. (2025)
Sauerkraut	Decrease	Production of lactic acid, γ-aminobutyric acid, D-phenyl-lactate and indole-3-lactate had a protective effect	Caco-2 cells	Wei and Marco (2025)

## 4 Short chain fatty acids and the intestine

Short-chain fatty acids (SCFAs), fatty acids with one to six carbon atoms, are the principal end products of anaerobic fermentation of complex and non-digestible polysaccharides by gut microbiota. The predominant SCFAs, acetate, propionate, and butyrate, are produced in the human colon in an approximate ratio of 60:20:20, respectively (Cummings et al., 1987; Martin-Gallausiaux et al., 2021). Their production is influenced by food composition, site of fermentation, and the composition of the intestinal microbiota. SCFAs, in particular butyrate, serve as energy sources for colonocytes and regulate intestinal homeostasis, metabolism, and immune responses (Salvi and Cowles, 2021; Correa-Oliveira et al., 2016).

**FIGURE 3:**
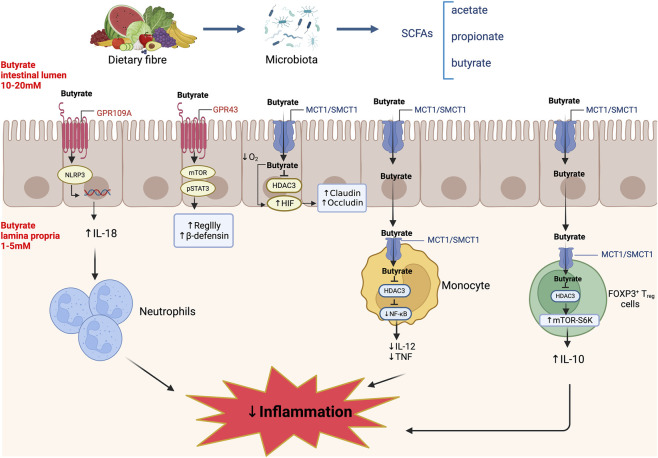
Butyrate regulates the integrity and function of the intestinal mucosal barrier through various mechanisms. In the intestinal lumen, butyrate reaches concentrations of between 10 mM and 20 mM and is a key energy source for intestinal epithelial cells. In columnar epithelial cells, butyrate signals via G protein-coupled receptors (GPR43 or GPR109A), monocarboxylate transporter 1/sodium-coupled monocarboxylate transporter 1 (MCT1/SMCT1) and histone deacetylase (HDAC) inhibition to activate the NLRP3 inflammasome and induce the downstream expression of IL-18, antimicrobial peptides and upregulating the expression of tight junction proteins claudin and occludin. SCFA induce neutrophils migration to inflammatory site and enhance their phagocytosis. It supports FOXP3^+^ expression and IL-10 secretion during the differentiation of regulatory T (Treg) cells. Butyrate inhibits the transcription of pro-inflammatory cytokines such as IL-12 and tumour necrosis factor (TNF) in monocytes and macrophages. Created in BioRender (https://BioRender.com/bktwbdh).

Among the SCFAs, butyrate has emerged as a crucial regulator of intestinal barrier integrity and immune modulation. As illustrated in Figure 3, it exerts its effects via activation of G protein-coupled receptors (GPCRs) including GPR41 (FFAR3), GPR43 (FFAR2), and GPR109A (HCAR2), which are expressed on epithelial cells and immune cells (Moniri and Farah, 2021; Sun et al., 2017). The superfamily of GPCRs is one of the largest families of proteins in the mammalian genome and shares a conserved structure composed of seven transmembrane helices (Fredriksson and Schioth, 2005). In parallel, butyrate also functions as a histone deacetylase (HDAC) inhibitor, influencing epigenetic gene expression (Davie, 2003). These dual mechanisms allow butyrate to modulate T cell responses, promote regulatory T cell (T_reg_) differentiation, induce antimicrobial peptide expression (e.g., β-defensins, RegIIIγ), and maintain epithelial barrier integrity (Isobe et al., 2020; Marinelli et al., 2019; Sun et al., 2017).

Butyrate contributes to the maintenance of colonic mucosal integrity and protection against disease progression in BALB/c mice with experimental colitis. It regulates cellular proliferation, apoptosis, and differentiation, thereby preserving epithelial homeostasis and reducing susceptibility to colitis and colorectal cancer (Donohoe et al., 2014). The importance of GPCR signalling in mediating these protective effects has been demonstrated in murine models. Mice deficient in GPR43 exhibit an exacerbated colitis-like phenotype, while administration of SCFAs, including butyrate, ameliorates disease in wild-type but not GPR43-deficient animals, underscoring the receptor’s essential role in SCFA-mediated intestinal protection (Masui et al., 2013; Smith et al., 2013). Beyond the intestine, butyrate also confers systemic benefits. In *db/db* mice, a model of Type 2 diabetes, butyrate improved intestinal barrier function and attenuated diabetic nephropathy-induced muscle atrophy. These effects were mediated via GPR43 activation and upregulation of the PI3K/Akt/mTOR pathway, which suppressed oxidative stress-induced autophagy in skeletal muscle (Tang et al., 2022a). These findings collectively support the concept that butyrate enhances intestinal barrier integrity and modulates inflammation via both local and systemic mechanisms, acting through key signalling pathways including GPCRs, HDAC inhibitors and epigenetic regulation.

### 4.1 SCFAs and TJ regulation

Among the SCFAs, butyrate exerts the strongest effects on epithelial barrier integrity, primarily by modulating TJ protein expression through multiple receptor-mediated signalling pathways, some of which are illustrated in Figure 3. *In vitro* and *in vivo* rodent studies reveal that butyrate enhances the expression of claudin-3 and claudin-4 in intestinal epithelial cells (Yan and Ajuwon, 2017), as well as claudin-1 and occludin (Fachi et al., 2019). SCFAs including butyrate also mitigate lipopolysaccharide (LPS)-induced barrier disruption by reversing decreases in TEER, reducing paracellular permeability, and restoring TJ morphology. These protective effects have been linked to inhibition of autophagy and NOD-like receptor protein 3 (NLRP3) inflammasome activation (Feng et al., 2018; Yan et al., 2022). Mechanistically, butyrate enhances TJ assembly via activation of 5′-adenosine monophosphate-activated protein kinase (AMPK). In Caco-2 cells, butyrate exposure activated AMPK, promoted the redistribution of ZO-1 and occludin to the TJ region, increased TEER, and decreased inulin flux. These effects were attenuated by AMPK inhibition, confirming the necessity of this pathway (Peng et al., 2009). Further insights by Miao et al. demonstrated that sodium butyrate elevates intracellular calcium through store-operated calcium entry, thereby activating AMPK in a calcium-dependent manner. Activated AMPK suppresses myosin light chain kinase (MLCK), reducing phosphorylation of myosin light chain 2 (MLC2) at Ser19 and facilitating TJ reassembly (Miao et al., 2016). Additionally, butyrate activates protein kinase C β2 (PKCβ2), increasing its phosphorylation at Ser660. Inhibition of PKCβ2 impaired TJ restoration, indicating that both AMPK and PKCβ2 signalling are essential mediators of butyrate-induced barrier enhancement (Figure 2) (Wang et al., 2023b).

Recent findings by Ornelas et al. have demonstrated that butyrate contributes to epithelial barrier tightness through the inhibition of iron-dependent prolyl hydroxylase enzymes, leading to the stabilization of hypoxia-inducible factor (HIF) (Ornelas et al., 2023). HIF is a master transcriptional regulator of multiple genes involved in erythropoiesis, angiogenesis, energy metabolism, antimicrobial, and anti-inflammatory processes (Hashimoto and Shibasaki, 2015). This effect plays a critical role in maintaining intestinal homeostasis. Butyrate also promotes oxygen consumption in intestinal epithelial cells, further enhancing HIF stabilization (Kelly et al., 2015). HIF, a key transcription factor, supports barrier function by upregulating claudin and occludin, thereby promoting epithelial repair and integrity (Pral et al., 2021). However, a potential clinical limitation of butyrate is that it is rapidly metabolized as an energy source thereby diminishing its availability to stabilize HIF. HIF stabilisers are emerging as an innovative therapeutic strategy for the treatment of IBD (Bhat and Rieder, 2022). However, a metabolite of butyrate, 4-mercapto butyrate, stabilizes HIF with a higher potency and has higher stability in intestinal epithelial cells compared to natural butyrate. *In vivo* studies with 4-mercapto butyrate confirmed this pattern for systemic HIF stabilisation in mice (Ornelas et al., 2023).

### 4.2 Role of butyrate in IBD and dysbiosis

Butyrate plays a multifaceted role in modulating immune responses, suppressing inflammation, and in preserving the integrity of the intestinal barrier (Hodgkinson et al., 2023; Stoeva et al., 2021). Sodium butyrate has gained particular attention as a potential therapeutic agent for IBD, especially UC (Recharla et al., 2023). Although findings from a recent meta-analysis of SCFAs in individuals with IBD reported reduced levels of butyrate and acetate, results across studies were somewhat inconsistent (Zhao et al., 2021). Nevertheless, substantial evidence implicates an impaired epithelial immune response to the gut microbiota in IBD pathogenesis (Saez et al., 2023). Notably, individuals with IBD show a depletion of butyrate-producing bacteria and diminished colonic butyrate concentrations compared to healthy controls, underscoring the immunomodulatory potential of butyrate (Hodgkinson et al., 2023). Inflammation itself may impair the production and utilization of butyrate. For example, TNF-α reduces butyrate consumption in intestinal epithelial organoids derived from individuals with IBD, suggesting that epithelial responsiveness to butyrate is diminished under inflammatory conditions (Ferrer-Picon et al., 2020). In addition, expression of monocarboxylate transporter 1 (MCT1), a key butyrate transporter, is downregulated in colonic epithelial cells from patients with Crohn’s disease, further compromising butyrate uptake (Ferrer-Picon et al., 2020). Importantly, emerging evidence suggests that microbial dysbiosis in IBD may not simply result from inflammation. In fact, healthy individuals with a high genetic risk for IBD exhibit lower abundance of *Roseburia* spp., a major butyrate-producing genus, implying that dysbiosis may precede clinical disease onset (Imhann et al., 2018). The beneficial effects of butyrate-producing bacteria are exemplified by *Faecalibacterium prausnitzii*, a commensal species with anti-inflammatory activity in murine models of IBD. These effects are partially mediated by butyrate, which inhibits histone deacetylase 1 (HDAC1), suppresses the pro-inflammatory IL-6–STAT3–IL-17 signalling axis, and promotes transcription of *Foxp3* to support T_reg_ cell homeostasis (Zhou et al., 2018). In T_reg_ cells isolated from mice, butyrate induces IL-10 production via increased acetylation of p70 S6 kinase and phosphorylation of ribosomal protein S6, indicating a requirement for mTOR–S6K pathway activation in IL-10 expression (Park et al., 2015). Additionally, in a mouse model involving the adoptive transfer of microbiota-specific T cells, butyrate-stimulated IL-10 secretion by Th1 and Th17 cells reduced colitis severity (Chen et al., 2019a). *In vitro*, butyrate and propionate also suppress LPS-induced maturation of human monocyte-derived dendritic cells, leading to reduced IL-12 secretion and diminished CD8^+^ T cell activation (Nastasi et al., 2017).

Butyrate exerts anti-inflammatory effects through inhibition of HDACs (Schulthess et al., 2019; Jiang et al., 2025; Korsten et al., 2023). HDACs are enzymes that remove acetyl groups from ε-N-acetyl lysines on histone proteins, leading to chromatin condensation and reduced gene transcription. Butyrate increases histone acetylation at promoter regions of inflammatory genes, thereby suppressing their transcription (Chang et al., 2014). Among HDAC isoforms, HDAC3 appears particularly important in mediating butyrate’s anti-inflammatory effects in macrophages (Jiang et al., 2025). In the lamina propria, where butyrate concentrations range from 1 to 5 mM, it modulates mucosal immune homeostasis by regulating immune cell differentiation, migration, and cytokine production. Butyrate is transported into monocytes via sodium-coupled MCT-1 (SMCT1) and MCT1. Once inside the cell, butyrate directly inhibits HDAC3, suppressing NF-κB activation and reducing the transcription of pro-inflammatory interleukin-12 (IL-12) and TNF-α (Jiang et al., 2025). *In vivo* evidence further supports these immunomodulatory actions. In a rat model of caecal ligation and puncture induced sepsis, sodium butyrate treatment attenuated systemic inflammation and preserved intestinal barrier function, in part by inhibiting NF-κB activation. These findings highlight butyrate’s potential as a therapeutic agent in polymicrobial sepsis and other inflammatory conditions (Yan et al., 2022; Fu et al., 2019).

### 4.3 Formulation strategies for oral administration of butyrate

The therapeutic potential of oral butyrate supplementation for managing gut inflammation has been investigated in preclinical models (Zha et al., 2020; Zhou et al., 2021; Lee et al., 2022) and clinical studies (Facchin et al., 2020; Pietrzak et al., 2022; Vernero et al., 2020). However, a major pharmacokinetic limitation is the rapid absorption of butyrate in the upper gastrointestinal tract, primarily the duodenum, resulting in minimal delivery to the colon when administered in conventional formulations. Furthermore, the clinical adoption of oral butyrate has been hindered by its unpleasant taste and odour. To address these challenges, various colon-targeted and encapsulated formulations have been developed to enhance the bioavailability of butyrate at distal intestinal sites.

One such product, Butyrose® Lsc® Microcaps (SILA® S.p.A.), uses lipophilic microencapsulation to enable the slow release of butyrate in lower parts of the intestine. In a double-blind, placebo-controlled pilot study, Facchin et al. evaluated the effects of Butyrose® (1,800 mg/day for 60 days) in 49 patients with IBD (Facchin et al., 2020). The treatment significantly shifted gut microbiota composition, increasing the abundance of butyrate-producing bacteria and thereby enhancing endogenous butyrate production, a change associated with improved gut homeostasis. Similarly, Butyrose® supplementation (1.1 g sodium butyrate/day for 90 days) was shown to beneficially modulate the gut microbiota of patients with symptomatic uncomplicated diverticular disease (SUDD). As dysbiosis is a recognized contributing factor in SUDD pathogenesis, the observed increases in microbial diversity and abundance of SCFA-producing taxa were accompanied by a significant reduction in abdominal pain symptoms (Tursi et al., 2025). Other formulation strategies have also demonstrated promise. Di Sabatino et al. showed that enteric-coated butyrate tablets (4 g/day) effectively reduced ileocecal inflammation and maintained clinical remission in patients with Crohn’s disease (CD) (Di Sabatino et al., 2005). More recently, Wang et al. developed butyrate-loaded polymeric micelles for targeted colonic delivery. In two murine models of colitis; DSS-induced colitis and CD45RB^hi^T-cell transfer colitis, butyrate micelles significantly improved intestinal barrier function and reduced disease severity (Wang et al., 2023a).

Vernero et al. conducted a 12-month pilot study assessing microencapsulated sodium butyrate (BLM) as an add-on therapy in patients with UC in clinical remission. Compared to patients receiving mesalamine (2.4 g/day) alone, those receiving combination therapy with BLM (1 g/day) demonstrated higher maintenance of clinical remission (83.3% vs. 47.6%), along with reductions in residual symptoms, inflammatory markers, and improvements in gut barrier protection and antioxidant capacity (Vernero et al., 2020). Collectively, these studies support the potential of formulated butyrate and butyrogenic compounds as adjunctive or alternative strategies for IBD therapy. While preclinical and clinical studies largely support its efficacy in UC and CD, controlled randomised clinical trials are necessary to determine optimal dosing, formulation, and its role as monotherapy or in combination with standard treatments. Future research should also explore synergistic effects of butyrate in combination with other SCFAs to maximize therapeutic efficacy in IBD management.

## 5 Medium-chain fatty acids and intestinal TJs

MCFAs are saturated fatty acids with chain lengths of 6–12 carbon atoms. Their role as intestinal PEs has been extensively studied since the 1990s, with seminal work by Artursson’s group on human Caco-2 monolayers (Lindmark et al., 1995; Lindmark et al., 1998; Anderberg et al., 1993). Among these, C_10_ and the C_8_ derivative, SNAC, have received the most attention due to their inclusion in proprietary delivery platforms and multiple clinical trials. C_10_ was initially formulated in GIPET™ (Gastro-Intestinal Permeation Enhancement Technology) by Elan Pharma (Dublin, Ireland) (Leonard et al., 2006) and later by Merrion Pharmaceuticals for oral peptide delivery (Walsh et al., 2011). It has also been used by Ionis Pharmaceuticals for oral delivery of antisense oligonucleotides (Maher et al., 2009b). SNAC and C_10_ remain the most widely studied PEs in oral peptide trials (Bohley and Leroux, 2024).

### 5.1 Sodium caprylate (C_8_)

C_8_ is a naturally occurring MCFA found in coconut oil, goat milk, and other dairy sources (Bennato et al., 2020). While traditionally used as a food emulsifier, it has demonstrated permeation-enhancing activity. C_8_ was the key PE in the development of Mycapssa®, an oral octreotide capsule formulation approved in 2020 by the US FDA for treating acromegaly (Melmed et al., 2015; 2020). This formulation employs Chiasma Pharma’s Transient Permeation Enhancer (TPE™) technology, which temporarily and reversibly modulates TJs, particularly by redistributing TJ proteins such as ZO-1 and claudins (Tuvia et al., 2014). Though the precise mechanism of action is not fully elucidated, evidence from coarse-grained molecular dynamics and high-content imaging suggests a concentration-dependent, multimodal transcellular mechanism, partly mediated by its surfactant-like properties (Hossain et al., 2020; Brayden et al., 2014). C_8_ is a less potent and efficacious PE than C_10_, (Brayden et al., 2014), suggesting that the efficacy of the TPE™ formulation may also depend on other components in the oily suspension (Brayden and Maher, 2021).

### 5.2 Sodium caprate (C_10_)

Sodium caprate, also known as sodium decanoate, is among the most studied intestinal PEs, with decades of research on its *in vitro* and *in vivo* mechanism of action as well, as extensive clinical trial studies in oral formulations of peptides and antisense oligonucleotides. Found in trace amounts in dairy fats, C_10_ is approved as a food additive in both the US and EU with no upper daily intake limits (Younes et al., 2018). It has been assessed in clinical trials by Merrion Pharma as an oral solid-dosage form (GIPET^TM^) for enhancing the absorption of Biopharmaceutical Class System III small molecules such as zoledronic acid and alendronate and macromolecules including insulin, desmopressin, and antisense oligonucleotides (Leonard et al., 2006). At concentrations of 50–100 mM *in vivo*, C_10_ enhances intestinal permeability with only mild and reversible mucosal damage (Wang et al., 2010; Berg et al., 2022). *In vitro*, low mM concentrations (e.g., 8.5 mM) reduce TEER and increase permeability across Caco-2 monolayers without compromising epithelial integrity, suggestive of a paracellular mechanism (Brayden et al., 2014; Lindmark et al., 1995). These concentrations affect both paracellular and transcellular pathways and are associated with membrane fluidisation and intracellular calcium signalling cascades involving phospholipase C, IP_3_, and MLCK, which trigger cytoskeletal contraction and TJ opening (Tomita et al., 1996; Feighery et al., 2008; Shimazaki et al., 1998).

A pivotal study by Krug et al. demonstrated that 10 mM C_10_ increases intestinal permeability primarily via the tricellular TJ route, by reversibly displacing tricellulin and claudin 5, leading to TEER reduction and increased paracellular transport of model hydrophilic markers (fluorescein, FITC-dextran) (Krug et al., 2013). Gleeson et al. demonstrated that C_10_ enhanced the permeability of the tripeptides, [^3^H]-IPP and [^3^H]-LKP across Caco-2 monolayers and rat jejunal tissue, even in the presence of the PepT1 substrate Gly-Sar. While Gly-Sar reduced both the antihypertensive effects and serum levels of the peptides, co-administration with C_10_ restored their bioavailability and blood pressure-lowering effects in Spontaneously Hypertensive Rats. These findings suggest that C_10_ may bypass PepT1 competition by promoting paracellular transport of IPP and LKP (Gleeson et al., 2018). Together, these results highlight the complexity of C_10_’s action and the limitations of using *in vitro* models to predict *in vivo* efficacy (Emeh et al., 2024).

C_10_’s performance in the GI tract may also be influenced by its interaction with bile salts and mixed micelles, areas still not fully understood regions (Maher et al., 2021). Nevertheless, there is now consensus that high local concentrations of C_10_ and payload are required for optimal permeation, which has favoured the design of immediate-release oral tablets (Maher et al., 2021; Tran et al., 2024) and spurred research into oral delivery devices (Ghavami et al., 2023). Notably, C_10_ is the PE in a (non-enteric) oral tablet of MK-0616, a macrocyclic PCSK9 inhibitor, which recently entered Phase III trials in 2024 for LDL cholesterol reduction (Ballantyne et al., 2023).

## 6 Chitosan and TJs

Chitosan is a linear, semi-crystalline polysaccharide derived from the deacetylation of chitin and consists of N-acetyl-D-glucosamine and D-glucosamine units (Aranaz et al., 2021). It is non-toxic, biodegradable, and widely used in the food industry and weight-loss products (Ahn et al., 2021; Quinn et al., 2022). Although chitosan has not received GRAS (Generally Regarded As Safe) status as a pharmaceutical excipient, it has been extensively investigated as an absorption enhancer in oral drug delivery (Singh et al., 2022; Tohidi and Aghaie-Khafri, 2023; Ryan et al., 2013; Vozza et al., 2018; Forde et al., 2023; Khalid Danish et al., 2022). Chitosan exhibits mucoadhesive properties and can transiently open intestinal epithelial TJs under acidic conditions, thereby enhancing paracellular transport (Takeuchi et al., 2005). Its mechanism is thought to involve interaction with apical integrins, activating downstream signalling pathways such as focal adhesion kinase and steroid receptor coactivators, resulting in TJ modulation (Hsu et al., 2012; Yeh et al., 2011).

Both *in vitro* and *in vivo* studies have demonstrated chitosan’s potential as a PE for hydrophilic drugs (Illum et al., 2002; Sonaje et al., 2010; Liao et al., 2010). Structural properties such as molecular weight and degree of substitution influence its performance. For instance, high molecular weight chitosan exhibits stronger mucoadhesion and paracellular permeability due to greater chain length and positive charge density (Schipper et al., 1996). Modified derivatives, such as oleic acid-conjugated chitosan, enhance solubility and improve the intestinal absorption of compounds like ferrous ions and insulin (Al-Remawi et al., 2022; Jaber et al., 2020).

Quaternised chitosan derivatives have demonstrated enhanced permeability at neutral pH. For example, 200-HPTChC53 (MW 200 kDa, 53% quaternisation) and 600-HPTChC65 (MW 600 kDa, 65% quaternisation) significantly increased paracellular transport in Caco-2 cells via TJ opening, with the latter also inhibiting P-glycoprotein-mediated efflux (Kowapradit et al., 2010; Wongwanakul et al., 2023). However, chitosan’s pH solubility profile limits its application in the small intestine, and its marine origin, batch variability, and limited GMP-grade availability restrict translational development (Li et al., 2025).

### 6.1 N-Trimethyl chitosan (TMC)

TMC is a quaternised chitosan derivative produced via reductive methylation, enabling solubility and absorption-enhancing activity in neutral pH environments where native chitosan is ineffective (Kotze et al., 1998). Its permeation-enhancing efficacy is positively correlated with its degree of quaternisation. In Caco-2 monolayers, TMC with 12% quaternisation reduced transepithelial electrical resistance (TEER) in a concentration-dependent manner and significantly enhanced insulin transport at pH 4.4 (Schipper et al., 1997; Kotze et al., 1997). TMC also increased the permeability of the hydrophilic markers, [^14^C]-mannitol and [^14^C]-PEG 4000, *in vitro*, with greater effects observed for lower molecular weight solutes (Kotze et al., 1998; Schipper et al., 1996). *In situ* rat studies using intestinal sacs and perfusion methods confirmed that absorption enhancement for macromolecules depended on both TMC concentration and quaternisation degree, with the best results observed at 48% (Jonker et al., 2002). These findings underscore the importance of charge density in modulating TJ permeability and drug absorption.

### 6.2 Thiolated chitosans (Thiomers)

Thiolated chitosans are emerging mucoadhesive and permeation-enhancing polymers with applications in peptide drug delivery. Their effectiveness stems from their ability to form disulfide linkages with mucosal surfaces and epithelial TJ proteins. For example, Sheng et al. developed insulin-loaded PLGA nanoparticles coated with TMC, which facilitated mucus penetration, TJ opening, and enhanced intestinal insulin absorption in diabetic rats (Sheng et al., 2015). Recent innovations include mercaptonicotinic acid activated thiolated chitosan (MNA-TG-chitosan), where disulfide stabilization improves polymer stability and absorption at acidic pH (Pratap-Singh et al., 2023). MNA-TG-chitosan demonstrated superior insulin permeability across human TR-146 buccal epithelial layers compared to unmodified chitosan, attributed to stronger TJ modulation and efflux pump inhibition. The disulfide moieties not only enhanced mucoadhesion but also increased TJ opening via protein tyrosine phosphatase inhibition, reducing occludin dephosphorylation. Chitosan and its derivatives (TMC, thiolated forms) offer promising strategies for enhancing oral delivery of peptides and hydrophilic drugs via TJ modulation. However, despite encouraging *in vitro* and preclinical data, clinical translation remains limited by challenges in formulation, solubility, manufacturing scalability and regulatory status. Continued development of modified derivatives and nanoparticle-based systems may help overcome these hurdles.

## 7 Amino acids and TJs

### 7.1 Glutamine

Glutamine is a conditionally essential amino acid that supports gut mucosal integrity, epithelial cell proliferation, and TJ maintenance for epithelial barrier function (Rao and Samak, 2012; Achamrah et al., 2017; Kuo et al., 2024). *In vitro*, glutamine supplementation upregulates claudin-1, occludin, and ZO-1 in Caco-2 monolayers, while glutamine deprivation or inhibition of glutamine synthetase increases permeability and reduces TEER (DeMarco et al., 2003; Li et al., 2004). This disruption is mediated via activation of the Phosphatidylinositol 3-kinase/protein kinase B (PI3K/AKT) pathway breakdown (Li and Neu, 2009). *In vivo*, glutamine protects against chemotherapy-induced intestinal injury. In a methotrexate (MTX)-induced mucositis model in rats, glutamine supplementation restored occludin, ZO-1 protein expression via ERK and NF-κB signaling pathways (Beutheu et al., 2013). Similar findings were observed under stress conditions, where glutamine pretreatment accelerated TEER recovery post-challenge and enhanced TJ protein expression via MAPK-dependent signalling (Li et al., 2004; Rhoads et al., 1997).

In piglets, glutamine improves gut barrier integrity during weaning stress, a critical period marked by mucosal vulnerability and inflammation (Shi et al., 2022). Several studies have shown the positive effects of glutamine as a nutritional supplement for piglets (Kuo et al., 2024; Schregel et al., 2022; Schulze Holthausen et al., 2022). Studies report improved intestinal morphology, barrier function, and performance outcomes with glutamine or glutamine-glutamate supplementation (Chalvon-Demersay et al., 2021; Schregel et al., 2022). Despite strong preclinical evidence, further dose-response and clinical trials are warranted.

### 7.2 Tryptophan

Tryptophan enhances intestinal barrier function through modulation of TJ proteins and immunoregulatory pathways. In porcine intestinal epithelial cells, L-tryptophan upregulated occludin, claudin-4, ZO-1, and ZO-2 (Wang et al., 2015). Liu et al. (2021a) demonstrated that tryptophan can improve intestinal epithelial barrier integrity and decrease inflammatory responses through the calcium sensing receptor (CaSR)/Ras-related C3 botulinum toxin substrate 1 (Rac1/phospholipase C gamma-1 (PLC-γ1) signalling pathway. In a DSS-induced porcine colitis model, tryptophan supplementation ameliorated clinical symptoms and reduced gut permeability (Kim et al., 2010). DSS was administered to piglets via intragastric catheter for 5 days followed by tryptophan administration at 80% of the daily recommended intake. It also suppressed pro-inflammatory cytokines (e.g., TNF-α, IL-6, IL-8, interferon (IFN)-γ, IL-12p40, IL-1β and IL-17) and enhanced apoptosis-regulating proteins (caspase-8, Bax), highlighting its potential for managing intestinal inflammation.

### 7.3 Citrulline

Citrulline is synthesized in enterocytes and serves as a functional marker of enterocyte mass and gut barrier integrity (Davila et al., 2013; Crenn et al., 2008). Plasma citrulline levels are decreased in conditions involving mucosal injury (e.g., villus atrophy, crypt necrosis), making it a potential biomarker of GI function loss (Papadia et al., 2010; Jäckel et al., 2020). Preclinically, citrulline protects against intestinal injury in models of sepsis, hypoxia, and colitis. It also conferred a protective effect on the intestinal mucosal barrier of mice against sepsis (Jianxin et al., 2023). Plasma levels of citrulline was lower in patients with acute GI infection-induced GI injury compared to patients with no infection. The results indicate that citrulline measurements, along with the acute GI injury assessment may have clinical potential in monitoring GI function and integrity in sepsis (Tyszko et al., 2023). Citrulline pretreatment improved gut barrier integrity and reduced bacterial translocation in isolated rodent ileal mucosae (Kaore et al., 2013). It serves as an indicator for early acute intestinal dysfunction in that a reduction in the number of intestinal epithelial cells is correlated with decreased serum citrulline levels (Shen et al., 2015). Citrulline also has roles in gut modulation, antioxidative and anti-inflammatory effects, protein synthesis, nitrogen homeostasis, blood pressure regulation, renal function, cardiac function, skeletal muscle function, vascular health, lipid and energy metabolism, arginine production, and thermoregulation (Breuillard et al., 2015; Allerton et al., 2018; Wong et al., 2016; Kaore et al., 2013). A study in human subjects reported a lowered number of inflammatory biomarkers following citrulline supplementation (Azizi et al., 2020). Its anti-inflammatory effects are characterized by reduction in pro-inflammatory cytokines, along with increase in anti-inflammatory cytokines in plasma. L-citrulline also exerted renal protective effects by reducing IL-1β and IL-12 but increasing IL-10 secretion in human proximal tubular cells (Romero et al., 2013). Several studies indicate efficacy of citrulline in serving as an arginine precursor and in nitric oxide production in acute or chronic inflammation (Lange et al., 2019; Schwedhelm et al., 2008). Overall, plasma citrulline is a functional (albeit unvalidated) marker of gut barrier dysfunction (Dey and Bhattacharjee, 2020), and lack of it has been associated with various intestinal diseases, including short bowel syndrome (Jeppesen et al., 2020; Jirka et al., 2019) and gastric ulcers (Liu et al., 2012).

Ho et al. (2022) investigated the potential synergistic effect of combining the probiotic, *Lactobacillus helveticus,* with citrulline to protect against damage induced by DSS in the mouse model of ulcerative colitis. L-citrulline enriched fermented milk with *L. helveticus* was administered by oral gavage daily for 12 days. It showed greater effects on protecting the damage induced by DSS, especially in ameliorating colonic permeability, and upregulating colonic ZO-1, occludin and claudin-1 expression. However, no protective effect was observed when treated with L-citrulline on its own.

### 7.4 Amino acid combinations

Glutamic and aspartic acid alone and in combination have beneficial and essential functions in nutrition and promote antioxidant effects in the intestine (Duan et al., 2016; Yin et al., 2015; Jiao et al., 2015). Low protein diets supplemented with glutamic and aspartic acid protected against oxidative stress-induced intestinal dysfunction in piglets (Chen et al., 2021). Manganese superoxide dismutase and glutathione peroxidase-1 are biomarkers of oxidative stress. Piglets challenged with hydrogen peroxide (H_2_O_2_), increased the ileal abundance of manganese superoxide dismutase along with jejunal and ileal expression of glutathione peroxidase-1 mRNA. Supplementation with glutamic and aspartic acid reduced both of these oxidative stress biomarkers in the H_2_O_2_-treated piglets. Catalase is an antioxidant enzyme which catalyzes H_2_O_2_ into water and oxygen. Piglets challenged with H_2_O_2_ downregulated catalase mRNA expression in the jejunum. However, supplementation of H_2_O_2 -_challenged piglets with glutamic- and aspartic acid increased expression of catalase mRNA.

Weaning stress in piglets also leads to lower nutrient absorption capacity and lower energy intake, resulting in body weight loss. Studies have shown positive effects of aspartate, glutamate, and glutamine as nutritional additives for piglets (Deng et al., 2023; Qi et al., 2020; Wang et al., 2019; Wang et al., 2022a). Aspartate, glutamate, and glutamine are the major energy fuels for the small intestine (Qi et al., 2020). Glutamate and aspartate are utilized in the intestine to yield adenosine triphosphate for enterocytes (Nakamura et al., 2013). They also play a pivotal role in intestinal homeostasis. One study has shown that supplementation with aspartate, glutamate, and glutamine under normal or low energy status can maintain the energy homeostasis of small intestinal mucosa in piglets by either replenishing the Krebs cycle or downregulating AMP activated protein kinase (Wang et al., 2022a). These amino acids also play key roles in regulating amino acid pool and protein synthesis, which are essential for gut epithelial proliferation, differentiation, and mucosa remodelling (Chen et al., 2021; Schregel et al., 2022). Another study found that supplementation of aspartate, glutamate, and glutamine in a normal or low energy level diet improved small intestinal barrier integrity in weaned piglets (Deng et al., 2023). Elevated levels of serum D-lactate and diamine oxidase (DAO) indicate intestinal mucosal damage and an increase in the intestinal permeability (Pinzer et al., 2018). DAO is a cytoplasmic enzyme found in the villus epithelial cells of the small intestine. Serum DAO levels have been evaluated as a potential marker of intestinal disease in a variety of disorders, including gut atrophy, ischemia, and inflammation (Fujiskai et al., 1993). The amino acids reduced gut permeability loss in weaning piglets. There was increased expression of occludin, claudin-1, and claudin-3 in the small intestine while the serum DAO and D-lactate levels were decreased in weaning piglets. In addition, supplementation with aspartate, glutamate, and glutamine increased the levels of proliferating cell nuclear antigen in the jejunum. These results suggest that supplementation with aspartate, glutamate, and glutamine improves intestinal barrier integrity by promoting intestinal epithelial cell proliferation and maintaining the structure of TJs.

Irradiation treatment inflicts acute injuries to the intestinal barrier which include epithelial cell death, villi shortening, crypt depletion, disruption of TJs and submucosal oedema (Nejdfors et al., 2000). Gupta et al. developed an amino acid-based oral rehydration solution which included ʟ-aspartic acid, ʟ-serine, ʟ-threonine, ʟ-tyrosine, and ʟ-valine. They tested the amino acid solution on radiation-induced changes of intestinal barrier function and epithelial TJs using a total-body irradiation (TBI) mouse model (Gupta et al., 2020). Irradiation induced ultrastructural changes of the intestinal TJs. TBI mice treated with the amino acid solution for 6 days improved intestinal epithelial barrier function in the ileal mucosa by increasing expression of barrier-forming claudins and removing pore-forming claudin-2 from epithelia TJs.

## 8 Zinc

Zinc is the second most abundant trace element in the human body and plays a pivotal role as a catalytic or structural cofactor in metalloenzymes and proteins. Zinc homeostasis is achieved through regulated uptake, distribution, and excretion, is essential for cellular function and systemic health. The small intestine is the central site for zinc absorption and excretion, making zinc homeostasis critical for maintaining the structure and function of the intestinal mucosal barrier (Adulcikas et al., 2018). Zinc absorption occurs via two main mechanisms: passive diffusion through ion channels and active transport mediated by specific zinc transporters. The two primary transporter families include the ZnT (SLC30A) family, which reduces intracellular zinc levels by promoting export, and the ZIP (SLC39A) family, which increases intracellular zinc by mediating import (Baltaci and Yuce, 2018; Huang and Tepaamorndech, 2013). ZIP4 is localized to the apical membrane of enterocytes and is the primary transporter for luminal Zn^2+^ uptake. Under zinc-deficient conditions, ZIP4 is upregulated at the membrane (Hashimoto et al., 2016; Dufner-Beattie et al., 2004; Weaver et al., 2007). Metallothioneins (MTs), cysteine-rich intracellular zinc-binding proteins, further regulate zinc storage, buffering, and trafficking within enterocytes. Under conditions of zinc sufficiency, ZIP5 on the basolateral membrane facilitates zinc efflux into the bloodstream, whereas ZIP14 also contributes to basolateral zinc transport but appears less sensitive to zinc status (Hashimoto et al., 2016; Dufner-Beattie et al., 2004; Weaver et al., 2007; Aydemir and Cousins, 2018). Excess zinc leads to the internalization and degradation of ZIP4 to prevent zinc overload.

Along with enterocytes, zinc is essential for the function of goblet and Paneth cells, which are key components of the intestinal mucosa and important for intestinal barrier integrity (Hollander and Kaunitz, 2020). Goblet cells secrete mucins such as MUC2, forming a protective mucus layer, while Paneth cells act as a zinc reservoir and produce antimicrobial peptides and epidermal growth factors (Knoop and Newberry, 2018; Ohashi and Fukada, 2019). Paneth cells also express Toll-like receptors (TLR-2, TLR-4), linking zinc homeostasis with innate immune responses and TJ regulation (Price et al., 2018). Intestinal TLR-2 signalling can control the TJs by maintaining ZO-1. ZnT2 is also critical in maintaining Paneth cell function, maintaining normal gut microbiota composition, and immune responses in mice (Podany et al., 2016). Other studies have reported that zinc supplementation increases the villus height and reduces the crypt depth in the mammalian small intestine (Zhu et al., 2017; Liu et al., 2014), as well as increasing the number of intestinal goblet cells (Maares et al., 2020) and MUC2 expression (Wen et al., 2018).

At the molecular level, zinc deficiency downregulates TJ proteins such as claudin-3 and occludin, leading to increased permeability and compromised barrier integrity (Camilleri, 2021; Zhong et al., 2010). Zinc supplementation restores these functions and activates the membrane-bound GPR39, initiating the Gαq–phospholipase C (PLC)–inositol triphosphate (IP3) signalling cascade. This leads to increased intracellular calcium, which activates calcium/calmodulin-dependent protein kinase kinase β (CaMKKβ), subsequently stimulating AMP-activated protein kinase (AMPK). The resulting kinase activation enhances TJ assembly and reinforces epithelial barrier integrity (Pongkorpsakol et al., 2019; Shao et al., 2017).

Zinc status directly influences the composition and function of the gut microbiota (Zackular et al., 2016; Vahjen et al., 2010; Liu et al., 2021b). Zinc deficiency reduces microbial diversity and promotes the overgrowth of zinc-tolerant pathogens, contributing to dysbiosis (Reed et al., 2015; Sauer and Grabrucker, 2019). Conversely, high-dose zinc supplementation in mice colonized with *Clostridium difficile* alters microbial composition and exacerbates disease severity by increasing toxin production and disrupting immune responses (Zackular et al., 2016). Zinc impacts microbiota through two key mechanisms: first by modulating host immune responses, such as macrophage-mediated bacterial clearance, and second, by serving as a cofactor for microbial growth and metabolic activity.

Zinc deficiency is associated with increased intestinal permeability, oxidative stress, and nitric oxide production, all of which can promote secretory diarrhea (Rodriguez et al., 1996; Cui et al., 1999; Bzik et al., 2012). In rodent studies, zinc deficiency increased expression of uroguanylin, a peptide promoting fluid secretion, and impaired absorption of triglycerides and proteins by altering chylomicron development and enterocyte peptidase activity (Blanchard and Cousins, 2000; David et al., 2022). Clinical evidence supports zinc’s therapeutic benefit in diarrheal diseases. Since 2004, WHO has recommended zinc supplementation alongside oral rehydration therapy for pediatric diarrhea (WHO/UNICEF, 2004). Zinc reduces the duration and severity of acute episodes and lowers recurrence risk in the months following supplementation (Bhutta et al., 1999; Medani et al., 2012; Fontaine, 2001). However, a Cochrane review concluded that benefits are more evident in children over 6 months old and in populations with high risk of zinc deficiency, with limited evidence for benefit in well-nourished or younger children (Lazzerini and Wanzira, 2016).

## 9 Flavonoids

Anthocyanins are water-soluble, coloured pigments belonging to the phenolic group of compounds and are responsible for the red, blue, and purple hues found in many plants, particularly fruits, flowers, and tubers. Structurally, anthocyanins are glycosylated forms of anthocyanidins, which are the aglycone counterparts. They are a subclass of flavonoids, a diverse group of polyphenolic secondary metabolites characterized by two aromatic rings linked via a three-carbon heterocyclic pyran ring. Common flavonoid subclasses include anthocyanins, flavones, flavonols, flavanones, isoflavonoids, chalcones, and aurones. Anthocyanins undergo various chemical modifications such as hydroxylation, methylation, glycosylation, and acylation, which influence their stability, colour properties, and biological activity (Houghton et al., 2021).

Anthocyanidins, particularly pelargonidin found in strawberries, have shown promising potential as intestinal permeation enhancers for macromolecules. Lamson et al. demonstrated that pelargonidin transiently and reversibly increases intestinal epithelial permeability in mice without inducing inflammation or tissue damage. In their study, oral delivery of insulin encapsulated with pelargonidin in a standard size 9 capsule to mice resulted in blood glucose reduction sustained for over 4 h, with pharmacological activity (PA) exceeding 100% compared to subcutaneous injection. These effects were associated with modulation of TJ and actin cytoskeletal dynamics and reversed within 2 h. Importantly, daily pelargonidin administration to mice for 30 days induced no detectable adverse histological or inflammatory effects, suggesting a potentially safe mechanism to facilitate peptide absorption (Lamson et al., 2022). However, limitations remain for pelargonidin being accepted as a PE with true potential, including the difficulty and cost of isolating pure pelargonidin, the lack of published data with other peptides, no validation in other laboratories, nor evidence from large animal models.

Beyond drug delivery, anthocyanins particularly those present in berries exhibit antioxidant, anti-inflammatory, and gut barrier supporting properties. In a high-fat diet (HFD) mouse model, anthocyanin supplementation (40 mg/kg body weight) reduced intestinal permeability, endotoxemia, and inflammation. This was accompanied by decreased plasma endotoxin levels, increased GLP-2 secretion, and upregulation of TJ proteins, occludin, ZO-1, and claudin-1, as well as enhanced MUC2 gene expression. FITC-dextran permeability was nearly doubled in HFD-fed mice but was reduced by anthocyanin supplementation, indicating improved epithelial barrier integrity (Cremonini et al., 2019).

Anthocyanins from *Lycium ruthenicum* Murray, a medicinal herb, have multiple bioactivities including antioxidant (Sadowska-Bartosz and Bartosz, 2024), anti-inflammatory (Peng et al., 2019), neuroprotective (Chen et al., 2019b) and anti-obesity (Tian et al., 2021) effects. Peng et al. reported that anthocyanins from *L. ruthenicum* attenuated neuroinflammation in a high-fructose diet-induced mouse model. This effect was mediated through the maintenance of intestinal barrier integrity and suppression of the TLR4 signalling pathway, as confirmed using *Tlr4* knockout mice. These findings suggest a gut–brain axis mechanism whereby anthocyanins modulate systemic inflammation via effects on the microbiota and epithelial barrier (Peng et al., 2023). *In vitro* fermentation of anthocyanins enhanced gut microbial diversity in patients with acute cerebral infarction, promoting the growth of beneficial taxa (e.g., *Bifidobacterium*, *Allisonella*, *Prevotella*) while suppressing potentially pathogenic genera (e.g., *Dialister*, *Megamonas*, *Clostridium*). These shifts were accompanied by increased production of SCFAs, indicating prebiotic-like effects (Qiu et al., 2024). Overall, anthocyanins isolated from *L. ruthenicum* Murray has similar effects to prebiotics and promotes the production of SCFAs by modulating gut microbes (Liang et al., 2024).

**FIGURE 4:**
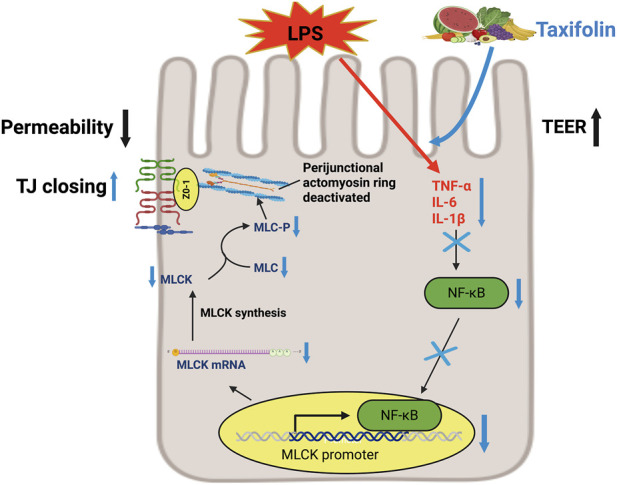
Schematic illustrating Taxifolin maintains intestinal epithelial tight junction integrity through inhibition of the NF-κB/MLCK signaling pathway, leading to reduced LPS induced inflammation-associated epithelial barrier dysfunction. Created in BioRender (https://BioRender.com/52h8n4c).

Taxifolin, a naturally occurring flavonoid found in plant-based foods like fruit, vegetables, wine, tea, and cocoa, attenuated lipopolysaccharide (LPS)-induced effects in Caco-2 cell monolayers by reducing secretion of the inflammatory markers, TNF-α, IL-1β, and IL-6 (Gong et al., 2022). Mechanistically, taxifolin suppressed LPS-induced activation of the NF-κB pathway, as indicated by decreased phosphorylation of NF-κB, along with reduced MLCK expression and MLC phosphorylation. Taxifolin improved epithelial barrier integrity by increasing transepithelial electrical resistance and reducing FITC-dextran permeability, while restoring the expression and continuous distribution of tight junction proteins, including claudin-1, ZO-1, and occluding (Figure 4). Overall, these findings suggest that taxifolin protects intestinal epithelial barrier function primarily through inhibition of the NF-κB/MLCK signalling axis. Overall, anthocyanins represent a promising class of food bioactives with multifaceted health benefits, particularly in enhancing intestinal barrier function and mitigating inflammation. Their potential in managing conditions such as IBD, metabolic syndrome, and neuroinflammation is supported by growing preclinical evidence. More research in animal and human trials, however, is needed to elucidate the relevant mechanisms of action.

## 10 Fermented foods

Fermentation was used historically as a food preservation process where sugars are broken down by bacteria and yeasts resulting in the production of antimicrobial metabolites (e.g., organic acids, ethanol and bacteriocins), which reduces the risk of contamination with pathogenic microorganisms. Plant-based fermented foods offer an underexplored reservoir of probiotic strains with therapeutic potential. Naturally fermented foods, such as kombucha, yoghurt, and kimchi, have gained a lot of attention as reports of potential health benefits in animal models and humans have emerged (Dimidi et al., 2019). During the fermentation process of these foods, biochemical reactions are triggered by multiple microorganisms that result in the release of vitamins, amino acids, enzymes, exopolysaccharides (EPS), SCFAs, organic acids, phenolic compounds, and bioactive peptides, among others, which provide potential benefits on human health (Dalile et al., 2019; Silva et al., 2020; Liu et al., 2025). The natural fermentation of dairy products has been recorded as far back as 10,000 BC. Elie Metchnikoff proposed the first ‘health claim’ for a fermented food in 1910 when he accredited good health and longevity to the consumption of fermented milk (Metchnikoff, 1907). Live cultures are found in not only yogurt, but also in Korean pickled vegetables called kimchi and sauerkraut. Fermentation can result in the generation and/or enrichment of a diversity of bioactive compounds, several of which have been associated with beneficial effects across various gastrointestinal conditions. Considerable advances have been made in the study of fermented foods, the microorganisms and bioactives they contain, and more recently, their impact on the human microbiome and gut health.

### 10.1 Kefir

Kefir is a traditional fermented beverage originating from the Caucasus and is produced by inoculating milk with kefir grains, which consist of symbiotic cultures of lactic acid bacteria (LAB), yeasts, and acetic acid-producing bacteria. The fermentation process yields a product rich in probiotics, bioactive peptides, and other beneficial compounds (Lopitz-Otsoa et al., 2006). Kefir has been extensively studied for its potential to modulate gut microbiota and alleviate inflammation, particularly in conditions such as metabolic syndrome, colitis, and colorectal cancer. Consumption of kefir may positively influence gut microbiota composition. In animal models, kefir supplementation has been linked to an increase in beneficial bacteria such as *Lactococcus*, *Lactobacillus* and *Bifidobacterium*, while reducing pathogenic species like *Clostridium perfringens*, *Firmicutes*, *Proteobacteria*, and *Enterobacteriaceae* (Hamet et al., 2016). Kefir also provided protective effects in Giardia intestinalis infections in C57BL/6 mice, likely through immune modulation (Franco et al., 2013). In dogs, kefir supplementation increased the ratio of LAB to *Enterobacteriaceae*, promoting a healthier gut microbiome (Kim et al., 2019). Furthermore, kefir has been shown to alter the activity of specific gut microbiota strains. *Lactobacillus kefiranofaciens* was isolated from kefir and can adhere to Caco-2 intestinal cells and inhibit the adherence of pathogenic *Salmonella typhimurium* and *Escherichia coli O157:H7* (Santos et al., 2003; Hugo et al., 2008; Huang et al., 2013). *In vivo* studies also support this mechanism; BALB/c mice challenged with *E. coli O157:H7* had reduced infection symptoms and less intestinal damage following pre-treatment with *L. kefiranofaciens M1* (Chen et al., 2013). This suggests that kefir, particularly its *Lactobacillus* strains, plays a key role in enhancing gut barrier function. Additionally, kefir has been found to increase SCFA production, including butyrate, a key metabolite involved in gut health (Choi et al., 2025). *Lactobacillus acidophilus* is among the most widely studied probiotic species and has been associated with diverse health benefits, including protective effects in gastrointestinal, metabolic, and inflammatory disorders. A specific strain, *L. acidophilus* LA1, has recently been shown to exert both protective and therapeutic effects on the intestinal epithelial tight junction (TJ) barrier (Haque et al., 2024). Tumor necrosis factor-α (TNF-α) induces a rapid and sustained increase in intestinal TJ permeability through transcriptional activation of the MLCK gene in intestinal epithelial cells (Ma et al., 2004; Ye and Ma, 2008). In Caco-2 cell monolayers and *in vivo* murine models, LA1 effectively attenuates TNF-α–induced TJ barrier disruption via a Toll-like receptor-2 (TLR2)–dependent mechanism that suppresses NF-κB p50/p65 activation. Crosstalk between the LA1/TLR2 and TNF-α/TNF receptor signalling pathways is mediated, at least in part, by inhibition of phosphoinositide 3-kinase (PI3K) activity at the level of IκB kinase-α (IKKα), leading to downstream suppression of enterocyte NF-κB p50/p65 signalling and consequent inhibition of MLCK gene expression.

Kefir’s therapeutic potential has also been demonstrated in various disease models. In an azoxymethane/DSS-induced colorectal cancer mouse model, kefir supplementation modulated gut microbiota composition, reducing the relative abundance of *Clostridium* and *Aspergillus*, while decreasing inflammatory markers and cancer cell proliferation (Zeng et al., 2021). Similarly, kefir’s anti-inflammatory properties have been demonstrated through its downregulation of pro-inflammatory cytokines (IL-6, TNF-α, IL-1β) and upregulation of anti-inflammatory cytokines (Albuquerque Pereira et al., 2023). These effects were also noted in a study on mice with alcoholic liver disease, where kefir supplementation led to decreased inflammatory markers and restored gut microbiota balance (Cui et al., 2024). In addition to its gut health benefits, kefir may also influence the gut–brain axis. A study on mice found that kefir administration increased catalase and superoxide dismutase levels, as well as SCFAs, including butyrate and propionate in the colon and brain, suggesting a protective role for kefir in both intestinal and brain health (Albuquerque Pereira et al., 2023). Similarly, a 12-week intervention with kefir in individuals with metabolic syndrome resulted in significant changes in microbiota composition, including an increase in Actinobacteria, which are important for maintaining gut homeostasis (Bellikci-Koyu et al., 2019). Similar results have also been supported by other studies (Ye et al., 2023). Overall, kefir holds promise as a therapeutic agent for improving gut microbiota composition, enhancing intestinal barrier function, and modulating immune and inflammatory responses. While studies in animal models seem to show promising results, clinical research is needed to better understand the underlying mechanisms and therapeutic potential of kefir in human health.

### 10.2 Sauerkraut

Sauerkraut, a traditionally fermented vegetable product, is rich in LAB with digestive, immunomodulatory, and antipathogenic properties. In traditional preparations, endogenous bacteria from cabbage initiate fermentation in an anaerobic brine environment, converting plant-derived carbohydrates into lactic acid along with carbon dioxide, acetic acid, and various bioactive compounds (National-Research-Council, 1992; Shahbazi et al., 2021). Fermentation involves dynamic shifts in microbial populations, with LAB such as *Lactobacillus plantarum*, *L. brevis*, and *L. sakei* dominating at various stages (Plengvidhya et al., 2007; Lee et al., 2011). *In vitro* studies highlight sauerkraut’s potential prebiotic effects. During simulated gastrointestinal digestion, low-salt sauerkraut exhibited high microbial survival rates of up to 72% and altered gut microbiota composition in porcine fecal fermentation models. This included an 85-fold increase in *Megasphaera* spp., a genus capable of producing vitamins, essential amino acids, and SCFAs (Chan et al., 2021; Shetty et al., 2013). LAB derived from fermented vegetables have been shown to exert immunoregulatory effects, generate bioactive compounds such as isothiocyanates, and inhibit pathogens via acidification and bacteriocin production (Touret et al., 2018; Shahbazi et al., 2021). Notably, some isolates demonstrate antimicrobial activity against pathogens such as *Listeria monocytogenes*, *E. coli* O157, and *Candida albicans*, suggesting a role for sauerkraut as a complementary therapy for gastrointestinal infections and disorders, including IBS (Touret et al., 2018; Yu et al., 2013; Jung et al., 2021).

Emerging human studies further support the health benefits of sauerkraut. Wei et al. demonstrated that fermented cabbage, unlike its raw counterpart, protected Caco-2 intestinal epithelial monolayers from inflammatory damage induced by IFN-γ and TNF-α. Soluble compounds formed during fermentation, including lactic acid, γ-aminobutyric acid (GABA), D-phenyl-lactate, and indole-3-lactate, contributed to this protective effect by increasing TEER and reducing paracellular permeability to FITC-dextran (Wei and Marco, 2025). Fermented vegetables are also gaining interest in sports nutrition for microbiota modulation. In a short-term intervention, 11 athletes consuming 250 g/day of organic pasteurized sauerkraut for 10 days exhibited alterations in gut microbiota composition, with an increased abundance of *Lachnospiraceae* and shifts in metabolic pathways related to cell wall and nucleotide metabolism (Karacic et al., 2024). However, findings are not uniform across populations. In a larger crossover study involving 84 healthy adults, daily consumption of 100 g fresh or pasteurized sauerkraut for 4 weeks produced no significant changes in overall gut microbial composition, as assessed by shotgun metagenomic sequencing (Schropp et al., 2025). Only pasteurized sauerkraut increased serum SCFA levels, whereas fecal SCFA concentrations declined following fresh sauerkraut consumption. This suggests that sauerkraut enhanced host absorption of SCFA since SCFA transporters are upregulated under healthy conditions. Serum SCFA concentrations may therefore serve as potential biomarkers of metabolic health, whereas elevated fecal SCFAs have been associated with conditions such as IBS and obesity. Collectively, these findings highlight the potential of sauerkraut and other fermented vegetables to support intestinal barrier integrity, modulate gut microbiota, and deliver health-promoting microbial metabolites. However, interindividual variability and product-specific factors (e.g., fresh vs. pasteurized, salt content) warrant further investigation in diverse human populations.

## 11 Conclusion

The complexity of gut barrier regulation involves crosstalk between epithelial cells, immune responses, and the microbiota. These relationships present a challenge in elucidating specific effects of individual food components, if present. Food-derived modulators of tight junctions are poised to play a significant role in gut health maintenance and disease prevention. The field is evolving rapidly, driven by a better understanding of gut physiology, microbiota interactions, and bioactive compound chemistry. Despite growing evidence supporting the beneficial effects of food-derived molecules on the maintenance and restoration of intestinal barrier integrity, several key challenges remain. One major limitation is the lack of standardized *in vivo* models and clinically relevant endpoints to assess barrier function, which impedes the translation of preclinical findings into therapeutic or preventive strategies. Additionally, the bioavailability, metabolic fate, and optimal dosing of many bioactive compounds remain poorly characterized, complicating their application in functional foods or nutraceuticals. The complexity of gut barrier regulation, which involves cross-talk between epithelial cells, immune responses, and the microbiota, also presents a challenge in isolating the specific effects of individual food components. Practical limitations of translating food-derived molecules into clinical therapies are to isolate pure molecules, to establish their potency and efficacy as individual or combined moieties in cell bioassays, to work out their pharmacokinetics in preclinical animal models, and then to manufacture them with excipients in pharmaceutical oral solid dosage forms at the correct dose levels for subsequent clinical testing. Looking ahead, future research should include a multidisciplinary collaboration across nutrition, medicine, and pharmaceutical sciences. Priority should be given to well-designed human intervention studies, integrate systems biology approaches, and explore delivery technologies that enhance the stability and targeted action of these molecules. Advances in organ-on-a-chip models, multi-omics analyses, and microbiota-host interaction studies are expected to deepen our understanding of how food components modulate the intestinal barrier. Ultimately, such insights could pave the way for precision nutrition strategies aimed at preventing or managing barrier-related disorders such as inflammatory bowel disease and support early-life or aging gut health where intestinal barrier integrity is especially vulnerable.
